# Perfusion Imaging in Autoimmune Encephalitis

**DOI:** 10.1155/2018/3538645

**Published:** 2018-05-06

**Authors:** Deepak Vallabhaneni, Muhammad Atif Naveed, Rajiv Mangla, Awss Zidan, Rashi I. Mehta

**Affiliations:** ^1^Department of Internal Medicine, SUNY Upstate Medical University, Syracuse, NY, USA; ^2^Department of Radiology, Shaukat Khanum Memorial Cancer Hospital, Lahore, Pakistan; ^3^Department of Radiology, SUNY Upstate Medical University, Syracuse, NY 13210, USA; ^4^Department of Neurology, SUNY Upstate Medical University, Syracuse, NY, USA; ^5^Department of Radiology, West Virginia University, Morgantown, WV, USA

## Abstract

Encephalitis is characterized by inflammation of brain tissue and has various infectious and noninfectious causes. CSF analysis and MRI usually reveal inflammatory changes although sometimes brain imaging may be normal. Autoimmune encephalitis is caused by antibodies against neuronal synaptic receptors, surface proteins, or intracellular proteins. In this case report, we present a 65-year-old female who presented with a fall and altered mental status. Workup for infectious etiologies was negative and MRI of the brain displayed focal restricted diffusion with corresponding T2-FLAIR hyperintensity involving gray matter structures, making the diagnosis unclear. CT perfusion of the brain demonstrated increased cerebral blood volume and cerebral blood flow in the left parietooccipital gray matter, with corresponding normal mean transit time. Following treatment failure with acyclovir, antibiotics, and steroids, the patient was found to be positive for GAD65 antibodies and diagnosed with autoimmune encephalitis. Symptoms markedly improved with plasmapheresis. Autoimmune encephalitis rarely causes restricted diffusion and this is the first case report to describe corresponding hyperperfusion on CT perfusion study.

## 1. Introduction

Encephalitis is characterized by inflammation of brain tissue, causing symptoms such as headache, fever, altered mental status, seizures, and/or focal neurologic deficits. CSF analysis and MRI usually reveal the findings of inflammatory changes although normal findings are not uncommon. Etiologies of encephalitis are diverse, but autoimmunity has been increasingly identified. Autoimmune encephalitis is caused by antibodies against neuronal synaptic receptors and surface or intracellular proteins [1]. Despite the severity of symptoms that such antibodies can entail, immunotherapy has been shown to be highly effective [2]. Clinical signs specifically pointing to autoimmune encephalitis include subacute memory loss, psychiatric symptoms, and sleep disturbance [3]. Conversely, infectious features such as fever, vomiting, and MRI contrast enhancement are rarely seen in autoimmune encephalitis [4]. As a diagnosis of exclusion, it is important to rule out infectious etiology as well as malignant, metabolic, and toxic etiologies before arriving at the diagnosis of autoimmune encephalitis [3]. Subsequently, a search for autoreactive antibodies ensues in both serum and CSF [3]. EEG findings may include slow activity focally or as background, with or without epileptiform discharges. On imaging, MRI can range from normal to T2-FLAIR scattered hyperintensities [3].

## 2. Case Report

**Figure 1:**
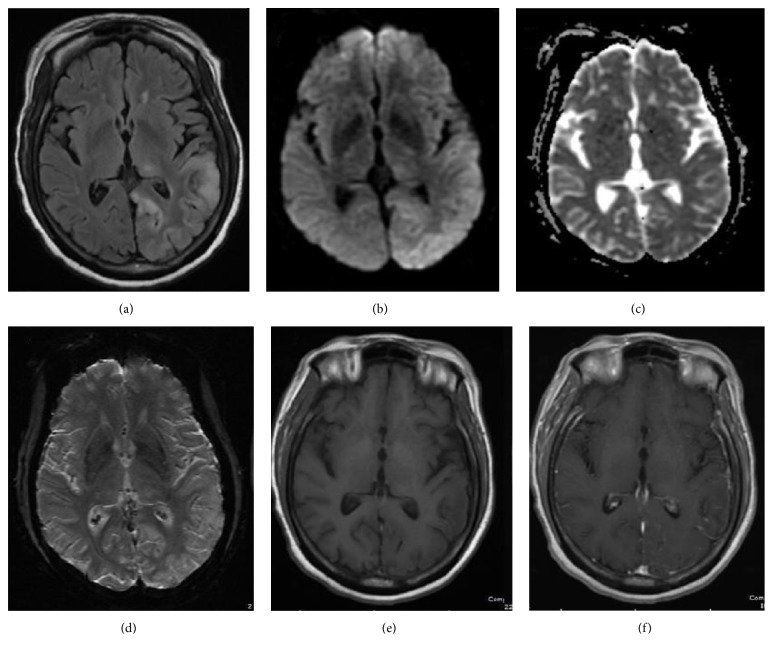
64-year-old female with confusion, right lower extremity weakness, and questionable encephalitis. Axial FLAIR MR image (a) shows increased signal intensity and gyriform swelling in the cortex and subcortical white matter of the left parietooccipital lobe and a focus in the left thalamus. Diffusion weighed (b) and attenuation diffusion coefficient, ADC, MR images (c) reveal mild decreased diffusivity. No susceptibility artifact or contrast enhancement is observed on axial susceptibility weighted image, SWI (d), and axial T1 weighted (e) and axial T1 weighted postgadolinium images (f), respectively.

**Figure 2:**
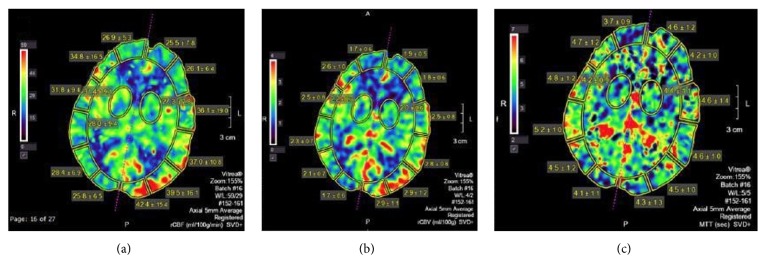
CT perfusion was performed 10 days after the initial MRI imaging. The postprocessed CT perfusion maps in axial plane at the level of the left parietooccipital cortical abnormality reveal increased cerebral blood flow (CBF) and cerebral blood volume (CBV) in the affected cortex on the left as compared to the normal brain parenchyma on the right. The time to peak (TTP) is decreased.

A 65-year-old female, with previous medical history of diabetes mellitus, stroke, hypertension, hypokalemia, and bipolar disorder, presented with gradually progressive subacute alteration of mental status manifesting as fluctuating awareness and impairment of cognitive functions including memory, language, and processing. Simultaneously, she developed motor deficits leading to repetitive falls and right hemiparesis. Spasticity with involuntarily posturing later ensued. Initial CSF analysis revealed mild monocytic pleocytosis, with no identified bacterial, viral, or fungal pathogens. The patient was monitored closely for convulsive and/or nonconvulsive seizures during the time of her admission by three-day video EEG monitoring, and three spot EEGs, all within 10 days of the CT perfusion, and none of them demonstrated the transformation of the epileptogenic discharges into seizures. There was no change in mental status or neurological examination to suggest emergence of seizures on the day of CT perfusion. MRI of the brain showed left parietooccipital cortical swelling with decreased diffusivity and abnormal hyperintense signal on T2- FLAIR sequences, suggesting encephalitic etiology (Figure 1). CT perfusion of the brain showed increased cerebral blood volume (CBV) and cerebral blood flow (CBF) and corresponding decreased time to peak (TTP) in the left parietooccipital cortex as compared to the contralateral normal brain tissue (Figure 2). Empiric treatment with steroids did not lead to significant improvement. Serum, and then later CSF, paraneoplastic epilepsy panel was positive for GAD65 antibodies with high titers (serum 1068 nmol/L, normal <= 0.02). The lab evaluation was performed at Mayo Medical Laboratories which suggest a cutoff of 20 nmol/L for major neurologic disease.

All other antibodies were tested negative in serum and CSF in a full antibody workup (Mayo panel). The search for a causative tumor by full-body CT scans at the time of the presentation and then one year later was negative. A concomitant elevation of Thyroid Peroxidase antibodies occurred transiently and then resolved. Immunomodulating treatment with plasmapheresis resulted in definite, yet incomplete, improvement. Subsequent CSF analysis revealed increased GAD65 antibodies 1.83 nmol/l, normal <= 0.02. Due to incomplete clinical improvement, immunosuppression with Rituximab was initiated. Five months later, the patient had recurrence of right-sided weakness and gait problems along with new right-sided sensory loss with MRI displaying worsening of the left parietooccipital lesion. At the time of this manuscript, the patient continues to show clinical improvement from her original presentation but with persistent significant disability.

## 3. Discussion

Autoimmune encephalitis can present in both adults and children [5]. The clinical manifestations are variable and may include prodromal symptoms, headache, altered mentation, seizures, and/or focal neurological deficits. The wide array of manifestations frequently leads to a diverse differential diagnosis with a myriad of investigative imaging studies. A variety of neuronal antibodies have been identified in autoimmune encephalitides. The vast majority of these encephalitides share some clinical and radiological features, although some uniqueness exists based on the location of the underlying immune response [6].

Glutamic acid decarboxylase (GAD) is an intracellular enzyme that is present at the terminal synaptic levels and catalyzes the synthesis of gamma-aminobutyric acid, the major inhibitory neurotransmitter in the CNS [7]. The anti-GAD antibodies typically cause a form of autoimmune encephalitis with classic temporal lobe lesions on MR imaging with the expected clinical findings of limbic encephalitis, and/or additional features of stiff person syndrome with early and prominent development of seizures [6].

This case demonstrates certain distinct clinical and radiological findings. First, this patient presented with extralimbic encephalitis based on the MRI findings, and the correlating EEG, which is atypical for GAD65 autoimmune encephalitis, which is frequently limbic in location [8]. Brain MRI showed cortical restricted diffusion which pathophysiologically signifies cytotoxic edema limiting water content in cells [9]. Second, the presence of hyperperfusion on CT perfusion exam of the brain with corresponding decreased diffusivity and T2-FLAIR hyperintensity in the cortex of the left parietooccipital lobe was observed. A very recent case report (in December 2017) has also found increased cerebral blood flow on arterial spin labelling in in anti-NMDA receptor encephalitis [10]. This case illustrates similar findings in anti-GAD encephalitis with CT perfusion. It has also been reported that perfusion imaging can demonstrate abnormality in autoimmune encephalitis even before the lesions are identifiable on conventional MRI sequences. The exact mechanism of focal increased perfusion in autoimmune encephalitis is not known but possibility of loss of cerebral vascular autoregulation has been suggested. In conclusion, this case demonstrates the utility of perfusion imaging in radiological diagnosis of autoimmune encephalitis, which may enable earlier diagnosis, more prompt therapy, and improved patient outcome.

## References

[1] Armangue T., Petit-Pedrol M., Dalmau J. (2012). Autoimmune encephalitis in children.

[2] Lancaster E., Martinez-Hernandez E., Dalmau J. (2011). Encephalitis and antibodies to synaptic and neuronal cell surface proteins.

[3] Kalman B. (2017). Autoimmune encephalitides: A broadening field of treatable conditions.

[4] Lancaster E. (2016). The diagnosis and treatment of autoimmune encephalitis.

[5] Dalmau J., Lancaster E., Martinez-Hernandez E., Rosenfeld M. R., Balice-Gordon R. (2011). Clinical experience and laboratory investigations in patients with anti-NMDAR encephalitis.

[6] Kelley B., Patel S., Marin H., Corrigan J., Mitsias P., Griffith B. (2017). Autoimmune encephalitis: pathophysiology and imaging review of an overlooked diagnosis.

[7] Pinal C. S., Tobin A. J. (1998). Uniqueness and redundancy in GABA production.

[8] Malter M. P., Helmstaedter C., Urbach H., Vincent A., Bien C. G. (2010). Antibodies to glutamic acid decarboxylase define a form of limbic encephalitis.

[9] Özbek O., Koç O., Paksoy Y., Aydin K., Nayman A. (2011). Epstein-Barr virus encephalitis: Findings of MRI, MRS, diffusion and perfusion.

[10] Sachs J. R., Zapadka M. E., Popli G. S., Burdette J. H. (2017). Arterial spin labeling perfusion imaging demonstrates cerebral hyperperfusion in anti-NMDAR encephalitis.

